# Modulating Chaperone-Mediated Autophagy and Its Clinical Applications in Cancer

**DOI:** 10.3390/cells11162562

**Published:** 2022-08-17

**Authors:** Virginie Hubert, Sebastian Weiss, Andrew Jackson Rees, Renate Kain

**Affiliations:** 1 Department of Pathology, Medical University of Vienna, 1090 Vienna, Austria; 2 Comprehensive Cancer Center, Medical University of Vienna, 1090 Vienna, Austria

**Keywords:** autophagy, cancer, chaperone, chaperone-mediated autophagy, KFERQ, LAMP-2A, lysosome, protein degradation

## Abstract

Autophagy is a central mechanism for maintaining cellular homeostasis in health and disease as it provides the critical energy through the breakdown and recycling of cellular components and molecules within lysosomes. One of the three types of autophagy is chaperone-mediated autophagy (CMA), a degradation pathway selective for soluble cytosolic proteins that contain a targeting motif related to KFERQ in their amino acid sequence. This motif marks them as CMA substrate and is, in the initial step of CMA, recognised by the heat shock protein 70 (Hsc70). The protein complex is then targeted to the lysosomal membrane where the interaction with the splice variant A of the lysosomal-associated membrane protein-2 (LAMP-2A) results in its unfolding and translocation into the lysosome for degradation. Altered levels of CMA have been reported in a wide range of pathologies including many cancer types that upregulate CMA as part of the pro-tumorigenic phenotype, while in aging a decline is observed and associated with a decrease of LAMP-2 expression. The potential of altering CMA to modify a physiological or pathological process has been firmly established through genetic manipulation in animals and chemical interference with this pathway. However, its use for therapeutic purposes has remained limited. Compounds used to target and modify CMA have been applied successfully to gain a better understanding of its cellular mechanisms, but they are mostly not specific, also influence other autophagic pathways and are associated with high levels of toxicity. Here, we will focus on the molecular mechanisms involved in CMA regulation as well as on potential ways to intersect them, describe modulators successfully used, their mechanism of action and therapeutic potential. Furthermore, we will discuss the potential benefits and drawbacks of CMA modulation in diseases such as cancer.

## 1. General Introduction

**Figure 1:**
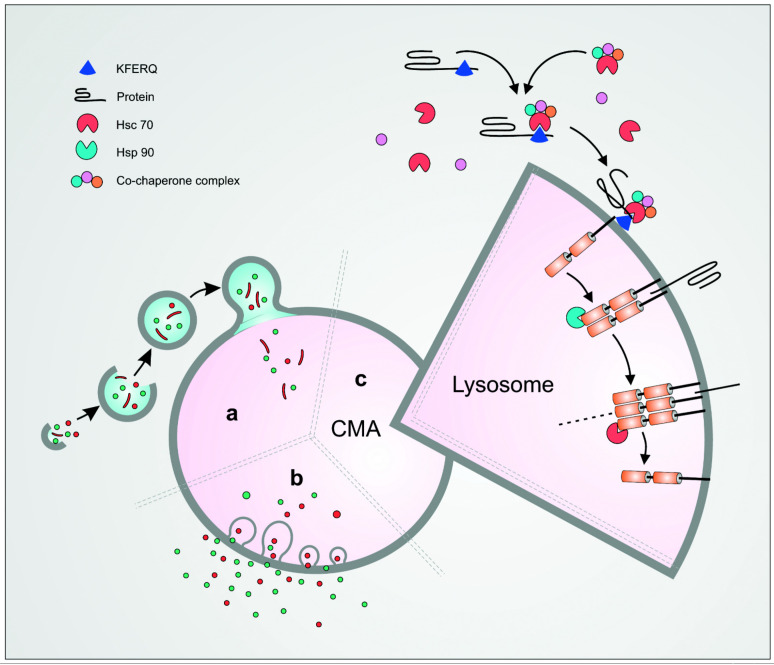
Schematic representation of the three main types of autophagy. Macroautophagy involves the sequestration of the cargo inside a double membrane vesicle—the autophagosome—followed by its fusion with the lysosomes (**a**). In microautophagy, an invagination of the lysosomal membrane occurs into which the substrate is engulfed (**b**). Chaperone-mediated autophagy (CMA) is a selective type of autophagy in which a protein bearing the KFERQ motif is recognised by the cytosolic chaperone Hsc70 and its co-chaperone complex. The complex is then targeted to LAMP-2A on the lysosomal membrane where it will unfold and translocate into the lysosome (**c**).

The term autophagy, from the ancient Greek autóphagos meaning ‘eating of self’, was introduced by Christian de Duve in 1963 to describe the formation of cytoplasmic vacuoles that imbibe intracellular proteins and organelles and deliver them to lysosomes [1].Now termed macroautophagy to distinguish it from other forms described, the central organelle in this process is the autophagic vacuole. Its characterisation provided the means to monitor macroautophagy in early studies [2] and its confirmation in all eukaryotic cells, including yeast [3]. This in turn facilitated the rapid delineation of the underlying molecular mechanisms and demonstration of the critical importance of macroautophagy for health, of both the individual cell and the organism [4] (Figure 1). The other two well-characterised forms of autophagy are microautophagy and chaperone-mediated autophagy (CMA). In the former, cytoplasmic material is imbibed directly into invaginations in the lysosomal and endosomal limiting membrane that are then pinched off and released as vacuoles to the lumen [5]. CMA, in contrast, is unique as substrate is not transported to lysosomes by vacuolar import. Instead, selected proteins that express a specific targeting motif bind to the ubiquitous cytoplasmic protein Hsc70 (heat-shock protein 70) and dock directly onto lysosomal-associated membrane protein-2A (LAMP-2A), its unique receptor in the lysosomal membrane, for import across the lysosomal membrane and degradation (Figure 1).

Evolutionarily, CMA is much younger than macroautophagy and until recently was thought to be restricted to birds and mammals. However, a LAMP-2A homologue has recently been identified in several fish species, and shown to participate in a CMA-like process [6,7]. Even so, lack of suitable model organisms such as worms, flies, or yeast, have severely constrained the dissection of the molecular mechanisms involved. Nevertheless, manipulation of LAMP-2 expression clearly demonstrated the importance of CMA for cellular health and aging through regulating homeostasis and responses to stress and disease, such as autoimmunity [8,9,10,11,12]. This renders CMA a potential target for therapeutic intervention and highlights the urgent need for specific modulation of CMA on a molecular basis. Here we review the current state of CMA biology, focusing on recent advances in the regulation of its activity; and describe the compounds presently available that modulate CMA, and their potential clinical use, especially in the context of cancer.

## 2. Mechanism of CMA

CMA was the first autophagic process pointing towards the degradation of intracellular components by the lysosome in a selective manner. Ribonuclease A (RNase A) was the initial CMA substrate identified by Fred Dice who monitored its degradation in human fibroblasts. The rate of degradation was enhanced after serum removal while these catabolic abilities were diminished in senescent cells [13]. Subsequently, his group characterised the key elements in the CMA pathway, including the identification of a pentapeptide sequence, Lys-Phe-Glu-Arg-Gln (KFERQ), required for targeting RNase A and other cytoplasmic proteins for lysosomal degradation [14]. This KFERQ motif was then found to constitute the bait that enabled heat shock cognate 71 kDa protein (Hsc70) to bind proteins targeted for CMA-mediated degradation [15]. Finally, LAMP-2A was identified as the receptor for the substrate/Hsc70 complex facilitating its import across the membrane and its degradation into the lysosomal lumen [16]. This established the essential stages of the CMA pathway and opened the way to a more detailed analysis of each of the steps involved and how they are controlled (Figure 1).

## 3. Targeting of Proteins for Degradation by CMA

The specific KFERQ sequence of RNase A is essential for binding Hsc70, but subsequent analyses have revealed that similar motifs in other proteins also facilitate binding. All of them are penta- or tetra-peptides with a consensus sequence that consists of one or two basic amino acids (Lys [K], Arg [R]) linked to one or two hydrophobic residues (Phe [F], Leu [L], Ile [I], Val [V]) and an acidic residue (Glu [E], Asp [D]), flanked by glutamine residues on either end [17]. In silico analysis identified KFERQ-like motifs in around 45% of human proteins, thus suggesting that they are common. Although often exposed on the surface they can be sometimes buried within the 3D structure of proteins which would correlate with a report that only 35% of cytosolic proteins extracted from human fibroblasts could be precipitated by antibodies specific to KFERQ [18]. Furthermore, acetylation or phosphorylation of specific residues can trigger Hsc70 binding and induce CMA, even in proteins without KFERQ-like motifs; this occurs in diverse situations, including high glucose concentration or IKK activation [18,19,20]. Similarly, cryptic Hsc70 binding motifs that are normally buried within the protein structure can be revealed under degradative conditions such as oxidative stress [21].

## 4. Hsc70 and Its Interaction with Target Proteins

Hsc70 is a constitutively expressed member of the HSP70 family of heat shock proteins, and has two domains: the substrate binding domain (SBS) and the nucleotide binding domain (NBD) with an intrinsic ATPase activity that powerfully affects target protein binding and release [22]. Hsc70 is a protein with multiple functions that, besides initiating CMA, include acting as a chaperone for nascent and unfolded polypeptides as well as partaking in clathrin-mediated endocytosis. The interaction of cytosolic Hsc70 and its KFERQ-motif bearing substrate is mediated by a cycle of ATP binding and hydrolysis with the ADP-bound form of HSC70 having increased affinity for the substrate. The ATPase activity itself is modulated by various chaperones including the Hsp70 interacting protein (Hip) which promotes the assembly of Hsc70 with Hsp40 and the protein substrate while Hsp40 stimulates the ATPase activity. Coupling of the substrate with Hsc70 can be altered by BAG-1 (Bcl2-associated athanogene 1 protein) which may act as a positive or negative regulator of Hsc70, a contrasting observation potentially due to the variable isoforms of BAG-2.

**Figure 2:**
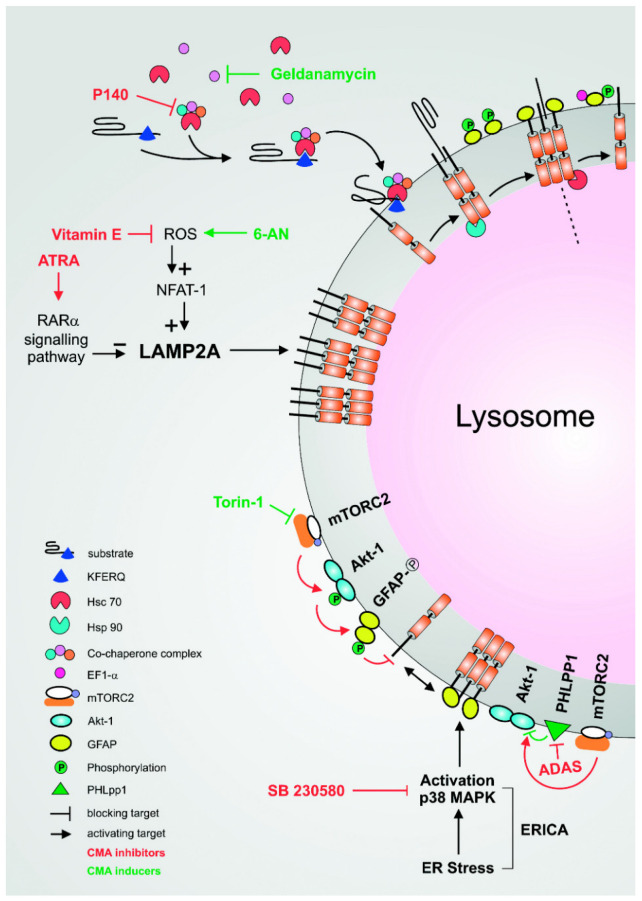
Inducers and inhibitors of CMA. Targeting the chaperone: One potential approach to modulate CMA involves the direct targeting of the chaperone such as Hsp90. Through the inhibition of the ATPase activity of Hsp90, Geldanamycin acts as an inducer of CMA. Conversely, the synthetic peptide p140 targets and hampers the chaperone function of Hsc70, acting as an inhibitor. Modulation of LAMP-2A levels: The expression of LAMP-2A is directly connected to the level of CMA. Reactive oxygen species (ROS) target the activation of transcription factor, NFAT-1 leading to its recruitment on the LAMP-2 promoter followed by an upregulation of its expression. Modulation of oxidative stress constitutes another potential approach to modulating CMA. Inducer of ROS, such as 6-AN, upregulates the level of CMA while vitamin E is a powerful antioxidant reducing or restoring its basal level. The mTORC2/PHLPP1/AKT pathway: The mTORC2/AKT1/PHLPP1 axis coordinates the dynamic assembly and disassembly of LAMP-2A into multimers. Torin-1 inhibits the function of mTORC2, therefore blocking the activation of AKT, promoting the formation of LAMP-2A multimers and, finally, inducing CMA. ADAS is a small molecule that inhibits the catalytic site of PHLPP1, thus blocking its phosphatase activity on AKT1. The phosphorylation of AKT1, which in turn phosphorylates the glial fibrillary acidic protein (GFAP), negatively regulates the dynamics of LAMP-2A assembly and inhibits CMA. The ERICA pathway: The ER-stress-induced CMA (ERICA) is a pathway connecting endoplasmic reticulum (ER) stress with CMA induction through the activation of p38 MAPK. The level of CMA is decreased in the presence of SB230580, an inhibitor of the activation of the p38 MAPK.

In a next step, the Hsc70/Hip/Hsp40 complex binds to the Hsp70-Hsp90 organising protein (Hop) which acts as an adapter between Hsc70 and its co-chaperone Hsp90, and is necessary to prevent protein aggregation [22]. Like Hsc70, the chaperone function of Hsp90 relies on the ATP and ADP/ATP exchange which is necessary for its interaction with the client proteins [23]. The macro-molecular complex consisting of the CMA target protein bound to Hsc70 and its co-chaperones is routed to the lysosomal surface where Hsc70 directly binds to the cytoplasmic tail of LAMP-2A [24]. The stability of this binding is augmented by the mitochondria-encoded peptide humanin, which is present at the cytosolic side of the lysosomal membrane where it interacts with Hsp90 and stabilises its binding to the substrate. This leads to a direct activation of CMA through multimerisation of LAMP-2A required for the translocation of the substrate into the lysosomal lumen [25,26] (Figure 2).

## 5. Assembly of the LAMP-2A/Hsc70/Protein Complexes on the Lysosomal Surface and Translocation into the Lysosomal Lumen

LAMP-2 is one of the most abundantly expressed proteins in the lysosomal membrane and has three domains: a heavily glycosylated luminal domain, a single trans-membrane domain and a short cytoplasmic tail. Three splice variants are expressed through alternate splicing of the LAMP-2 gene during transcription (LAMP-2A, B and C). They share an identical luminal and homologous trans-membranous domain but have different cytoplasmic domains. LAMP-2A is the only isoform involved in CMA and is unique as its cytoplasmic tail contains four positively charged residues [27] that are required for binding the Hsc70/protein complex to initiate CMA. LAMP-2A is in fact the only lysosomal receptor for the complex and its multimerisation is required for the translocation of the substrate [25].

Under resting conditions, LAMP-2A monomers are concentrated in cholesterol/glycosphingolipid-rich domains (lipid rafts) [28]. Following its exit from the lipid-enriched domain after induction of CMA, LAMP-2A is assembled into homotrimers forming multimeric channels that allow the interaction with Hsc70 and the protein substrate. It is subsequently linearised and translocated through the LAMP-2A channels to the lysosomal lumen for degradation.

To date, the molecular mechanisms triggering the formation of the LAMP-2A multimer complexes and the translocation of the substrate through the lysosomal membrane remain not fully understood [22,29]. Two chaperones related to CMA have been implied to play a critical role in this dynamic process, namely lysosomal Hsc70 (Lys-Hsc70) and Hsp90 (Lys-Hsp90). The functions of Lys-Hsc70 and Lys-Hsp90 are distinct from those of cytoplasmic Hsc70 and Hsp90 which are critical for the early stages of CMA. While Lys-Hsc70 promotes the disassembly of LAMP-2A and its conformation into the monomeric form, the interaction of Hps90 with the multimeric form of LAMP-2A within the lumen of the lysosome stabilises the channel during the conformational transition [25,30]. The multimeric LAMP-2 channels are, in addition, stabilised by the glial fibrillary acidic protein (GFAP). Conversely, its binding to the elongation factor 1-α (EF1α) and subsequent phosphorylation of GFAP after translocation of the substrate through the channel promotes its dissociation from LAMP-2A. Following the separation of the LAMP2A/GFAP complex LAMP-2A dissociates into the monomeric form that can be either degraded or recycled into lipid micelles [25].

The phosphorylation of GFAP is also mediated by the mTORC2/PHLPP1/AKT pathway during which the multimerisation and thus the assembly of LAMP-2 is inhibited directly at the lysosomal membrane early during the induction of CMA [31]. mTOR is a serine/threonine protein kinase that forms the catalytic subunits of two different proteins, the mTOR complex 1 (mTORC1) and 2 (mTORC2). Unlike mTORC1, mTORC2 is rapamycin insensitive and mainly phosphorylates AGC kinases including AKT [32]. mTORC2 acts as a negative regulator of CMA through the phosphorylation of AKT1 which in turn phosphorylates GFAP. The inhibitory effect of mTORC2 can be counteracted by the phosphatase PHLPP1 that, following CMA induction, is recruited to the lysosomal membrane. This inhibits AKT activation and leads to the dephosphorylation of GFAP which can now bind to LAMP-2A multimers and thus stabilises the channel and promotes CMA. Interestingly, many components of this finely tuned process (GFAP, EF1α and AKT1) bear a CMA-targeting motif themselves and are degraded, at least partially, by it. This motif may be used for loop regulation as well as a method of targeting to lysosomes [31].

The p38 mitogen-activated protein kinase (p38 MAPK) directly promotes the formation of the multimeric forms of LAMP-2A by phosphorylating it under endoplasmic reticulum (ER) stress. The ER constitutes a central component in the folding and assembly of secreted or membrane proteins that, properly assembled, leave the ER to the Golgi for further processing [33]. This tightly controlled process ensures that it meets and does not exceed ER capacity. However, aberrantly folded proteins or excessive protein synthesis because of physiological demands may result in the accumulation of unfolded proteins. This leads to ER stress, and the unfolded protein response (UPR) is an intracellular transduction pathway activated in response to the burden of unfolded proteins to restore cellular homeostasis. Activation of the UPR involves at least three distinct pathways, among them the PERK (PKR-like ER protein kinase)-dependent activation [33]. The link between macroautophagy and UPR is well established, however, the effect of ER stress on CMA remained until recently poorly described. In 2017, Li et al. reported for the first time a new signalling pathway connecting ER stress with CMA activation and named it ERICA for ‘ER-stress-induced CMA’. Using different ER stressors including treatments with protein transport, Ca^2+^ pump inhibitor, glycosylation suppressor or reducing agents, they observed PERK-dependent activation. This led to the recruitment of the mitogen-activated protein kinase 4 (MKK4) on the lysosomal membrane and the consecutive activation of a pool of lysosomal p38 MAPK (p38 mitogen-activated protein kinase). Lysosomal p38 MAPK directly phosphorylates LAMP-2A in positions T211 and T213, causing conformational changes of the molecule, its accumulation on the membrane in the multimeric form and activation of CMA. The specificity of this new pathway might constitute a very promising therapeutic approach to selectively target CMA [34].

## 6. LAMP-2A—The Lysosomal Receptor for the Hsc70/Protein Complex and a Central Player in CMA

The abundance of LAMP-2A in the lysosomal membrane is rate-limiting in CMA, however, its expression is predominantly regulated through changes in its dynamic distribution and turnover rather than de novo synthesis, although the latter can increase under certain conditions, such as mild oxidative stress [28].

Only LAMP-2A monomers are susceptible to cleavage, and degradation and one mechanism that has been described to regulate LAMP-2A levels is cleavage within the lipid microdomains of the lysosomal membrane. Under resting conditions, LAMP-2A monomers are concentrated in cholesterol/glycosphingolipid-rich domains (lipid rafts) [28] where the cytosolic tail is susceptible to enzymatic cleavage. This results in the release into the lysosomal lumen and potential degradation. In turn, LAMP-2A levels in the lysosomal membrane are decreased and CMA regulation is down. Exclusion of LAMP-2A from the lipid rafts prevents its proteolytic cleavage and allows the formation of LAMP-2 multimers required for the uptake of CMA substrate [35]. These changes are reflected in the half-life of LAMP-2A, estimated at around 38 h in resting fibroblasts with an increase to 77 h during starvation-induced CMA [36]. It follows that a generalised increase in lysosomal membrane cholesterol, such as it occurs during aging or in the presence of hyperlipidaemia, increases lipid raft-associated LAMP-2A and consequently its turnover. This leads to a reduction of its availability and finally a decrease of CMA [35]. Conversely, pharmacological reduction of cholesterol increases CMA and potentially could be a strategy to combat CMA-linked aging disorders [35]. A second form of regulation relies on its distribution between the membrane and the lysosomal lumen. Under conditions of CMA activation, intact LAMP-2A, found in the lysosomal lumen and associated to lipid micelles, can be recruited to the lysosomal membrane.

Under specific conditions such as oxidative stress, LAMP-2A levels can be directly modulated through the regulation of its transcription. This has been reported in T cells whose activation is associated with the mitochondrial generation of reactive oxygen species necessary for the activation of the nuclear factor of activated T cells-1 (NFAT1) and IL-2 production. Following activation, NFAT1 is recruited to the LAMP-2 promoter resulting in LAMP-2A up-regulation and consequently CMA activation. Binding of NFAT1 to the promoter is abolished after treatment with cyclosporin, a calcineurin inhibitor. CMA induction appears to also play a crucial role in the maintenance of activation-induced T-cell response as it leads to degradation of the ubiquitin ligase Itch and the calcineurin inhibitor Rcan-1. Three CMA targeting motifs have been identified in these proteins whose functions as negative regulators of TCR activation is well established [37]. Another pathway linking CMA modulation and LAMP-2A transcription is the retinoic acid receptor alpha (RARα) whose abolition is linked to a higher level of CMA. This increase of CMA is directly associated with a reduction of macroautophagy highlighting the opposing effect of RARα signalling on autophagic pathways. All-*trans*-retinoic acid (ATRA) is a potent activator of RARα that successfully reduces CMA in response to starvation thus confirming the inhibitory effect. This decrease has been associated with reduced LAMP-2A mRNA levels and may potentially explain the reduction of CMA activity [28,38]. The mechanism behind the activation of RARα and its ability to modulate CMA remains not fully understood but might result from a combination of multiple effects and will require further investigations [38]. Moreover, a recent study highlights the potential of the nuclear receptor corepressor 1 (N-Cor1), a protein able to repress the RAR signalling pathway. The ratio of N-Cor1/RARα expression directly correlates with CMA level, its increase being associated with a higher level of CMA. Interestingly, in patients suffering from retinitis pigmentosa, a progressive retinal disease leading to blindness, the ratio of N-Cor1/RARα was lower than in controls and associated with a decreased level of CMA, highlighting its potential for modulation [39].

Currently, there is no evidence that targeting LAMP-2 to the lysosomes could modulate CMA under physiological conditions. However, defective trafficking has been identified in selected pathologies including Parkinson’s disease (PD) and in the lysosomal storage disease cystinosis (caused by cystinosin deficiency). In PD, a mutation of the vacuolar protein sorting 35 (vps35) leads to an impaired endosome to Golgi retrieval of LAMP-2A [40] while in cystinosis the alteration of LAMP-2A trafficking is correlated with reduced expression of the GTPase Rab11 and the Rab7 effector RIPL, likely part of the transport machinery. Up-regulation of these molecules was able to rescue LAMP-2A trafficking at cellular level [41].

## 7. Visualising CMA

In 1956, Clark and Novikoff reported for the first time the presence of mitochondria embedded within vesicles that contain lysosomal enzymes. These sequestering vesicles, first called ‘dense bodies’ because of their ultrastructural morphology, were later termed autophagosomes. These double membrane vesicles contain partially digested material and ultimately fuse with lysosomes for degradation of their contents-known as macroautophagy. Later studies described the selective import and degradation of proteins in lysosomes, termed CMA, and identified its essential components, including LAMP-2A and Hsc70 [15,16]. However, unlike macroautophagy, the molecular characterisation of CMA was more challenging as specific ultrastructural features are absent which hindered the development of methods assessing and quantifying the processes involved [6,7].

Changes in CMA were initially detected through monitoring of CMA components such as LAMP-2A by immunoblotting. Abundance of this protein often correlates with changes of CMA activity but remains insufficient to firmly establish changes of this process. Another approach is to quantify the amount of ‘CMA-active’ lysosomes, determined by the co-localisation of Hsc70 and LAMP-2A on the lysosomal surface in proportion to the whole lysosomal pool. These methods, however, rely on the level of CMA markers that are highly variable, both between cell lines and individual cells, and their use in comparative studies is limited as they do not yield absolute numbers [42]. The correlative analyses should therefore always be complemented by functional assays, such as the degradation of radiolabelled long-lived proteins. It is measured by monitoring the release of free radiolabelled amino-acids into the media in the presence or absence of lysosomal protease inhibitors. Technically more challenging methods directly measure the uptake of CMA substrates by isolated lysosomes. However, these methods present some limitations when applied to tissue samples or in live-cell imaging that requires novel approaches recently developed, such as fluorescent reporters or photo-switchable constructs allowing to visualise CMA [43].

The first CMA reporter construct was developed by the group of Cuervo who fused the KFERQ motif of the RNAse A to a monomeric fluorescent protein (photoswitchable cyan fluorescent protein, or photoactivable mCherry protein). The photoconversion allows to easily visualise ‘CMA-active’ lysosomes as puncta with a limited background but also to monitor the degradation rate of the reporter [44]. Another approach was described in 2012 and is based on the fusion of the CMA substrate GAPDH with a Halo tag. The tag molecule can be labelled at neutral pH by brief incubation with a fluorescently labelled ligand where they stay covalently bound even after translocation into the lysosome. However, under acidic conditions binding between the tag and its ligand does not occur, which confirms the translocation of cytoplasmic proteins into the lysosomes. Using this method, Seki et al. successfully monitored CMA at single-cell level. As Hsc70 contributes to microautophagy, this method potentially monitors both of these processes. In order to distinguish the two processes, the Halo tag method has been combined with si-RNA-mediated knockdown of molecules specific for CMA, such as LAMP-2A, or microautophagy, such as TSG10, which has been successfully established by Sato et al. in 2016 [45,46]. The first transgenic animal model ubiquitously expressing a KFERQ-Dendra reporter to monitor CMA was developed and described in 2020. It appears to constitute a reliable experimental model to monitor basal and inducible CMA, and unlike in vitro assays, this model does not require photoswitch or activation as the cells contain considerably lower amounts of labelled protein in the cytoplasm. This reduces background fluorescence, and the binding of labelled substrate to LAMP-2A can be directly visualised and CMA monitored in living animals using intra-vital two-photon imaging [47]. Moreover, this mouse model provides the ground for a large range of methods to study CMA, including unfixed or fixed tissue sections and organotypic cultures of primary cells isolated from this animal (Table 1). Considering the role of CMA in several human diseases, this new tool provides a promising approach to monitoring its pathway and pathological changes over time and to evaluating potential pharmacological interventions (Table 1).

## 8. CMA Pathways and Their Pharmacological Modulation

### 8.1. The mTORC2/PHLPP1/AKT Pathway

One point of action at an initial level of CMA regulation lies at the lysosomal membrane where the mTORC2/AKT1/PHLPP1 pathway coordinates the dynamic assembly and disassembly of LAMP-2A into multimers.

The tricyclic benzonaphthyridinone inhibitor Torin1 was developed in 2010 and initially described as a phosphorylation inhibitor of mTORC1 and mTORC2 [48]. Through the competition with ATP at the catalytic site of mTOR, Torin-1 inhibits the kinase-dependent function of mTORC1 and mTORC2 and therefore blocks the activation of AKT. Subsequently, LAMP-2A multimers are formed, which promotes the uptake of CMA substrates—an effect not seen following treatment with rapamycin. Torin1 acts predominantly by enhancing CMA from basal levels, whereas only minor changes are observed once it has been induced that occur only within the last 4 h of stimulation (Figure 2). mTORC2 can only be increased after CMA induction in primary and non-cancer cell lines while in cancer cell lines Torin-1 does not further increase the level of CMA. Torin1 also confers increased resistance to oxidative and genotoxic stress, making this molecule a very interesting candidate for therapeutic use. At a concentration of 250 nM, the effect of Torin1 was more pronounced on mTORC2 than on mTORC1 whose specific inhibition by rapamycin did not alter CMA activity [31].

1-Amino-9,10-dioxo-4-(3-sulfamoylanilino)anthracene-2-sulfonic acid (ADAS) is a small molecule selectively inhibiting the catalytic site of PHLPP1 and therefore blocking its phosphatase activity on AKT1. Increased levels of phosphorylated AKT1 decrease CMA activity in response to serum starvation in a dose-dependent manner; however, higher concentrations of ADAS are required to affect basal CMA levels (Figure 2). ADAS has also been reported to decrease CMA levels following induction after oxidative stress. Although ADAS appears to be a specific inhibitor of CMA, publications reporting the selective use of this molecule remain limited [31]. However, in osteoarthritis where PHLPP protein phosphatases are abnormally abundant, the use of ADAS slowed down the degradation of articular cartilage in a mouse model with no severe side effects being reported which seems encouraging to extend its use also to other diseases [49].

### 8.2. The RARα Signalling Pathway and ATRA

Signalling via the RARα pathway is known to directly inhibit CMA (Figure 2). The development of synthetic derivatives of ATRA tightly and stably interacting with this receptor allows to neutralise the inhibitory effect in CMA. The magnitude of this antagonist effect likely results from the strong interaction between the synthetic compounds and its receptor, resulting from the introduction of mutations within the aromatic ring and residues of the *trans*-retinoic acid. Furthermore, the addition of groups such as -CN, -NH, or -B further enhances their reactive properties. Unlike the RARα antagonist BMS614, these ATRA derivatives specifically induce CMA but do not have an effect on macroautophagy, thus confirming their selectivity. Some of the compounds in combination bind the RARα simultaneously and, cumulatively, further increasing the activation of CMA. Thus, also the defence against oxidative stress and proteotoxicity was increased in cells treated with the synthetic compound prior to exposing them to the oxidant paraquat.

A recent publication by Gomez-Sintes et al. describes the generation of new molecules capable of selectively activating CMA as a result of further improvements of the synthetic derivatives previously described [38,39]. Through their conformation, these molecules display extensive hydrophobic interaction with the RARα-binding pocket and stabilise the recruitment of its co-repressor N-Cor1. This unique mechanism allows to specifically stabilise RARα in an inactive conformation preventing its switch to an active form necessary to recruit ATRA. Moreover, these molecules were shown to affect only a very specific subset of RARα-related genes, some of them being linked to the CMA networks. Two of these promising molecules have been tested on mice where they showed activation of CMA. Using a mouse model of RP, their administration was shown to upregulate CMA in the retina and was associated with a reduction of disease progression [39]. The specificity of these retinoid acid derivatives combined with the absence of toxicity their therapeutic potential as a specific CMA inducers in conditions such as aging or neurodegenerative disorders [38].

### 8.3. The ERICA Pathway

ERICA or ER-stress-induced CMA is a signalling pathway connecting ER stress to CMA through the activation of the lysosomal p38 MAPK, a protein involved in a broad range of functions including cellular response to inflammatory and stress response, cell growth and survival as well as cell differentiation [50]. One molecule targeting this pathway is the specific p38 MAPK inhibitor SB230580, and treatment of IMR 90 cells significantly reduces CMA induction (Figure 2). Its use has been suggested for the treatment of joint degeneration and pain due to osteoarthritis, as it enhances the constitutive level of apoptosis in cytokine-deprived eosinophils [51,52]. Although SB230580 appears to be a specific inhibitor of CMA, further studies investigating ERICA as well as its broader effect are necessary to firmly establish its specificity [53].

### 8.4. Hsp90

Hsp90 is a molecular chaperone found both in the cytoplasm where it takes part in the formation of the co-chaperone complex of CMA and in the lysosomal lumen where it stabilises the LAMP-2A multimers.

Geldanamycin is an anti-tumour antibiotic originally discovered in Streptomyces hygroscopicus, directly binding the *N*-terminal ATP binding domain of Hsp90. It inhibits Hsp90’s ATPase activity [54] and thus disrupts its interaction with client proteins leading to their degradation via the proteasome pathway. Among the client proteins are key oncogenic factors involved in the regulation of cell growth and differentiation which explains the rising interest in them as therapeutic targets [55]. Treatment with Geldanamycin has been shown to increase the level of CMA both in cells and in isolated lysosomes without affecting the level of macroautophagy [53] (Figure 2).

Unlike most molecules targeting the ATPase activity of Hsp90, the oxazoline analogue of apratoxin (Oz-apraA) inhibits Hsp90 activity by enhancing the interaction of its client proteins with Hsc70, thus inducing their degradation through CMA [23].

### 8.5. Hsc70

Hsc70 is one of the central components of CMA targeting cytosolic substrates to LAMP-2A via the KFERQ targeting motif. Lys-Hsc70 is also found in the lysosomal lumen where it partakes in the disassembly of LAMP-2A multimers. A synthetic 21-mer peptide that contains a phosphoserine residue at position 140 (P140) has been developed from the spliceosomal U1-70K small nuclear ribonucleoprotein. It targets the nucleotide-binding domain of Hsc70 thus hampering its chaperone function, but also blocks the shuttling of Hsc70 from the nucleus to the cytoplasm under stress conditions, which therefore remains sequestered in the nucleus. As a consequence, the cells lose their ability to survive a second ‘hit’, such as inflammatory stimuli [56].

In MRL/lpr lupus-prone mice, P140 binds to, and decreases the level of constitutively overexpressed Hsc70 and significantly enhances the survival of the animals. LAMP-2A, also overexpressed in these mice, is similarly downregulated after treatment with P140, which confirms an inhibition of both, LAMP-2A and Hsc70 in CMA. Interestingly, P140 also binds the major histocompatibility complex class II (MHCII) molecules and decreases their overexpression in MRL/lpr mice. Consequently, reduced levels of CMA in B cells alter the processing of the antigens as well as their loading onto MHCII and thus decreases the activation of auto-reactive T cells (Figure 2). In patients suffering from systemic lupus erythematosus, P140 significantly reduces the disease activity index [57]. Results of an early phase II clinical study were published in 2008 and showed improvement for these patients without significant clinical or biological adverse effects [57]. A large Phase 3 clinical trial was initiated in 2021 to test this inhibitor licensed under the name Lupuzor in lupus patients. [58,59,60]. The ability of P140 to regulate excessive CMA in vivo as well as to restore alterations of lysosomal defects in a pro-inflammatory context has also been recently demonstrated [61].

### 8.6. LAMP-2A

Reduced levels of CMA are common in aging and directly associated with a decrease in LAMP-2A stability and assembly. Increasing transcription of LAMP-2A in old transgenic animals restores LAMP-2A levels to those of young animals and increases CMA [62]. However, targeting LAMP-2A in CMA is limited because of its involvement in other processes, such as macroautophagy, where it is required for the fusion of the autophagosomes with the lysosomes [63]. Moreover, and as exemplified by the severe phenotype observed both in LAMP-2 deficient mice [64] as well as in patients suffering from Danon’s disease associated with a loss of LAMP-2 function, a therapeutic approach modulating LAMP-2 seems not practical [65].

Interestingly, in B cells, overexpression of LAMP-2C inhibits CMA by compromising the binding of Hsc70 to its substrate [66]. However, a potential reason of this inhibition might arise from a decrease of LAMP-2A in the lysosomal membrane that has been potentially overloaded with exogenously expressed LAMP-2C.

### 8.7. Oxidative Stress

CMA activity is detectable in all mammalian cells and although its basal level greatly differs between tissues and cell types, it is consistent between individual mice and among animal strains [44,47]. Striking differences have also been observed in the basal CMA level of cell types based on the organ they originate from, and variations observed between different cell types are a consequence of their ability to degrade specific proteins. In hepatocytes, for example, oxygen and nutrient gradient correlate with differential levels of basal CMA while these differences are abolished under conditions of total absence of nutrients [47]. Consequently, up-regulation of CMA follows a wide variety of stimuli such as starvation, oxidative stress, lipid challenge, or hypoxia [21,53,67,68].

Several factors are associated with oxidative stress such as obesity, high-fat diet, use of tobacco, pollution or metabolic disorders, promoting the accumulation of oxidised proteins, and finally altering CMA levels [69,70]. Oxidative stress correlates in particular with an increased level of LAMP-2A. Its modulation constitutes a potential approach to altering CMA as illustrated by 6-aminonicotinamide (6-AN), a monocarboxylic acid amide and powerful inducer of oxidative stress. 6-AN inhibits the glucose 6-phosphate dehydrogenase which in turn decreases the level of NADPH, leading to the production of ROS associated with an increased level of CMA (Figure 2) [21,71]. In contrast, the vitamin E succinate (VES) is a powerful antioxidant that, by reducing reactive oxygen species, can partially restore the initial level of CMA (Figure 2) [72]. Aging, a physiological state combining both metabolic and neurodegenerative changes, is similarly associated with a reduction of LAMP-2A levels that follows alterations in the lipid composition of the lysosomal membrane. Hence, accumulation of oxidative material within almost all cell types and tissues leads to the reduction of CMA [21,73,74].

In addition to these well-known effects, novel pathways with the potential for pharmacological intervention of CMA have emerged over the past years [21,53,67,68].

### 8.8. Cross-Talk between Macroautophagy and CMA

Macroautophagy and CMA are two important types of autophagy that are intimately connected despite their significant differences. Although both involve independent pathways, extensive cross-talk and compensatory mechanisms between the mechanisms are required to maintain cellular homeostasis. This is illustrated by the constitutive activation of CMA following the impairment of macroautophagy [75], while persistent blockage of CMA leads to constitutive activation of macroautophagy [76]. Spautin, an autophagy inhibitor, illustrates some aspects of this cross-talk. It inhibits the ubiquitin-specific peptidases (UPS) 10 and 13 that promote the degradation of the VPS34 PI3 kinase complex. This results in the inhibition of macroautophagy and, in parallel, in progressive increase of CMA.

The ability of each process to act as a back-up for the other pathway firmly establishes their existence, however, theses compensatory mechanisms remain not fully understood. More recently, it has been suggested that this cross-talk is cell-type specific and does not occur in all cells [47]. However, in some cell types, such as neurons, it appears to play a critical role as the compensatory mechanisms might lead to excessive or inappropriate activation of the other pathway and potentially to cell death or development of brain tumours [77].

## 9. CMA and Cancer

CMA is a mechanism central to the development, progression and resistance of cancer cells. Although in healthy cells CMA displays anti-tumour properties by preventing malignant transformation [78], its pro-tumorigenic functions take over in transformed cells. This dual role of CMA in a context-dependent manner is an essential component in the switch from anti-oncogenic to pro-oncogenic properties [79,80]

### 9.1. Anti-Oncogenic Roles of CMA in Healthy Cells

Decrease of CMA, first described as a hallmark of aging, directly correlates with an increased risk for many cancers. Despite some variations, this decline has been reported in most cell types and tissues and has been linked to decreased LAMP-2A stability due to modifications in the membrane lipid composition. In order to establish a clear role of CMA in the development of cancer, young and old mice with selective hepatic CMA inhibition were developed. These animals displayed a higher incidence of spontaneous hepatic tumours with age [81], which is partially attributed to the selective regulation and reduction of key proto-oncogenic proteins such as the mouse double-minute 2 homologue (MDM2) and the translational-controlled tumour-associated protein (TCTP) [82].

Liver-specific LAMP-2A-deficient mice also exhibit changes in proteasis affecting the protein quality control, which in turn promotes malignant transformation. While in young mice other proteolytic pathways compensate for the loss of CMA activity, this ability declines with age which explains the more severe phenotype reported in older animals [81]. The deficiency of LAMP-2A has also been associated with the abrogation of calreticulin exposure on the cell surface following stress. Calreticulin translocates to the cell surface under conditions of cell stress or immunogenic cell death, where it acts as a receptor for phagocytic cells [83]. This mechanism is engaged in the presentation of tumour antigens and in the anti-cancer cytotoxic T-cell-specific response, also called immunogenic apoptosis. However, the detailed analysis of this process remains to be elucidated [84,85].

CMA is also involved in the degradation of proto-oncogenes, as illustrated by c-MYC which is degraded by the protein phosphatase 2A (CIP-2A), itself being a CMA substrate. Blockage of CMA leads to an increase of CIP-2A followed by the accumulation and stabilisation of the oncogene MYC [78]. Similarly, degradation of the paired-box protein PAX2, a transcription factor playing a crucial role in cell proliferation and apoptosis, is regulated through CMA [86].

Conversely, cancer cells can also modulate CMA in the host cells to abolish their anti-oncogenic function. This has been observed, for instance, in glioblastoma where the interaction between the host pericytes and the glioblastoma leads to a massive oxidative burst. This in turn strongly upregulates CMA in the pericyte leading to the acquisition of immunosuppressive function in response to their interaction with glioblastoma cells. Finally, this precludes T-cell activation and tumour clearance, and promotes tumour survival [87].

### 9.2. The Roles of CMA in Malignant Neoplasias

#### 9.2.1. Pro-Oncogenic Role of CMA in Cancer Cells

In cancer cells, the anti-tumour properties of CMA become pro-tumorigenic by allowing the cells to sustain the increased metabolic requirement. While the level of macroautophagy varies depending on stage and type of cancer, CMA is consistently upregulated in numerous cancer cell lines [88]. In hepatocellular carcinoma, decreased levels of LAMP-2A affect tumour cell viability, tumour growth and recurrence [89]. In gastric cancer, the level of LAMP-2A expression in the tumour has been suggested as a promising biomarker for prognosis [90].

While the pro-tumorigenic functions of CMA in cancer cells are the subject of intensive investigations, the contribution of CMA in the transformation process, as well as its activation in cancer cells, remains poorly understood. CMA upregulation appears to take place shortly after tumour transformation and is mostly induced by well-known regulators of CMA and intrinsic to the tumour microenvironment such as oxidative stress, lack of nutrients and hypoxia [91].

#### 9.2.2. Glycolytic Capability

The transformation from healthy to cancer cells involves alterations of the cell metabolism including a switch to anaerobic glycolysis necessary for energy production independent of the presence of oxygen. This process, known as Warburg effect, is a typical feature of cancer cells and its maintenance relies on CMA as demonstrated for melanoma and lung cancer [79,92]. In these cancers, inhibition of CMA promotes the stabilisation of p53, a powerful anti-oncogenic molecule which in turn downregulates the transcription of glycolytic enzymes [88]. In other types of cancers, the level of glycolytic enzymes is directly regulated by CMA illustrating its direct role in the regulation of glycolysis. Among the enzymes involved in this switch from oxidative phosphorylation to anaerobic glycolysis is the embryonic M2 isoform of pyruvate kinase (PKM2). After exposure to a high concentration of glucose, PKM2 is glycosylated, resulting in decreased activity and degradation through CMA. Exposure to an acetylated mimetic mutant of PKM2 prevents this lysosomal degradation and promotes cell proliferation and tumour growth [19].

Hexokinase-II (HKII) is another enzyme involved in glucose metabolism and is recognised as a key factor for tumour initiation and maintenance. Under healthy conditions the HKII binds glucose, thus preventing its degradation through CMA by burying its KFERQ motif in the glucose-HKII bound structure. In the absence of glucose, the KFERQ motif of HKII is exposed and accessible for binding and degradation via CMA causing downregulation of some metabolic pathways and potentially cell death [93]. In cancer cells, the phosphorylation of HKII at Thr473 by Proviral Insertion in Murine Lymphomas 2(PIM2) prevents its degradation by CMA and increases both its stability and activity. This leads to enhanced glycolysis and an increase of glucose starvation-induced autophagy, finally promoting tumour growth [94].

#### 9.2.3. Cell Cycle and Proliferation

Alteration of cell deaths and aberrant cell survival constitute one of the major hallmarks of cancer. NF-κB is a key transcription factor found in the cytoplasm and kept inactive through its interaction with the inhibitory molecules IκB, a substrate for CMA [95]. IκB plays a key role in cell survival both under healthy and pathological conditions, while aberration of its signalling pathway is directly linked to numerous forms of malignancy. Moreover, this transcription factor is also involved in the regulation of autophagic process, as illustrated by the autophagic induction following the activation of the pro-tumorigenic NF-κB. These illustrates the existence of a reciprocal cross-talk between autophagy and NF-κB as well as its importance in suppressing or promoting cancer pathogenesis [96].

Several molecules involved in the control of the cell cycle are regulated by CMA, among them the tumour suppressor p73, capable of inducing cell cycle arrest and apoptosis. The nerve growth factor (NGFR) directly binds p73, suppressing its transcriptional activity and acting as a powerful oncogenic inhibitor of p73 by promoting its degradation via CMA. NGFR is upregulated in several types of cancers such as glioblastoma or breast cancer, enhancing transformed cell survival and negatively affecting p53, a pro-apoptotic transcription factor [97].

Another link between CMA and the cell cycle occurs with the Rho family GTPase 3 (RND3), an anti-proliferative protein degraded by CMA in gastric cancer cell lines. These regulators induce cell cycle progression when downregulated, whereas their upregulation arrests the cell cycle at the G0/G1 phase. RND3 also reduces MYC expression and its transcriptional activity. This transcription factor is a key regulator of the cell cycle and acts on numerous crucial genes such as cyclins, cyclin-dependent kinase (cdk) and EF2 transcription factor [78,89].

The hypoxia-inducible factor1 (HIF-1) is another transcription factor that negatively controls DNA replication under hypoxic conditions. One of the subunits of the heterodimer HIF-1, namely HIF-1α, is a CMA substrate whose degradation is regulated by CDK1 and CDK2. Interaction of HIF-1α with core components of the CMA machinery has been reported while overexpression of LAMP-2A decreased the HIF-1α level. Similarly, hypoxia-induced CMA in cancer cells leads to a reduction of HIF-1α as part of a negative feedback loop [98].

#### 9.2.4. DNA Damage Response

In addition to metabolic pathways and cell cycle regulation, CMA is also involved in DNA repair mechanisms and in the re-entry of the cell into the cycle following DNA repair. This process relies on the degradation of checkpoint kinase 1 (CHK1). CHK1 is a CMA substrate that upon site-specific phosphorylation is activated and therefore able to regulate DNA damage-induced cell cycle arrest. After repair, re-entering of the cell into the cycle takes place while activated CHK1 is degraded via CMA. Failure to regulate the degradation of CHK1 leads to a persistent activation and increase genome instability as reported in cancer cells [99].

## 10. CMA Modulation in Cancer

To date, there are many mechanisms that remain open or controversial with regard to the ‘Janus-faced’ role of CMA in healthy and cancer cells. Under physiological conditions, CMA plays a critical role in the regulation of cellular mechanism such as cell metabolism and function through the degradation of key regulatory factors. Any potential dysregulation of CMA quickly promotes the development of malignancy, turning this process into a powerful pro-tumorigenic instrument. However, the timeline of this transition from protective to harmful remains poorly understood, as does its direct contribution to the transformation into carcinogenic cells. An additional challenge also resides in the difference of CMA regulation observed between transformed vs. healthy cells. These unknowns make the development of efficient inhibitors very challenging although targeting differential regulatory mechanisms may constitute an excellent therapeutic approach to selectively altering cancer cells without affecting the healthy ones.

## 11. CMA, a New Target in Cancer Therapy?

Molecules modulating CMA in clinical user are to date very limited. For instance, Spautin, its analogue quizartinib (AC220), FLT3 inhibitors or A70 present promising results for the sensitisation of cancer cells to autophagy inhibition in vitro [93,100,101]. However, they lack specificity, and Spautin in particular has shown cardiac toxicity prohibiting its use for in vivo or even clinical studies.

Our understanding of the pathways involved in the physiological regulation of CMA remains still very limited and therefore renders the development of targeted chemical compounds difficult. In addition, many of the actors involved in CMA are also crucial for other cell functions which often lead to a high level of toxicity associated with the use of these compounds (e.g., inhibitor of lysosomal protease or inhibitor of protein synthesis). Finally, the activity of some of these molecules has been shown to arise from the compensatory mechanisms developed by the cells and leading to high variability between cell types [53].

## 12. Conclusions and Perspectives

Over the past decades, major breakthroughs in our understanding of the CMA process as well as CMA substrates, pathways and involvement in pathological disorders have been made. However, in comparison to other autophagic pathways, knowledge is still limited and many challenges remain to be tackled.

For instance, LAMP-2A was originally thought to be specific to mammals and birds, limiting CMA to these species. However, the identification of a LAMP-2A homologue in several fishes suggests that LAMP-2 actually appears at the root of the vertebrate lineage [6,7]. Considering the importance of CMA in a broad range of functions, this raises the question whether LAMP-2-like sequences remain unidentified in some species or whether there are other receptors for CMA that act in a similar manner. This was initially suggested following the observation of a steady-state level of CMA substrates in the cortex or hippocampus of LAMP-2-deficient mice. The absence of accumulation of CMA substrates under such conditions highlights the need to further investigate the potential additional factors or receptors within different cell types or tissues [9].

Additional complexity arises from the close interplay between macroautophagy and CMA. As described in this review, many modulators of CMA have been shown to also alter macroautophagy, either simultaneously or sequentially. For this reason, it has become essential to understand the molecular mechanisms and actors involved in this cross-talk in order to develop selective inhibitors or inducers.

Nowadays, the absence of selective chemical modulators of this pathway still constitutes an important obstacle in CMA research and therapeutic modulation. Most CMA actors are also involved in numerous other and essential cellular processes often leading to a significant level of toxicity. The most unique CMA component, namely LAMP-2A, shares a high level of homology with the two other known splice variants LAMP-2B and LAMP-2C, which renders it difficult as target.

Finally, a multitude of questions remains on the dual role of CMA as anti-oncogenic in healthy but pro-tumorigenic in cancer cells. Both function and regulation of this process appear to differ upon transformation, and the events mediating the switch from low to high CMA levels, their contribution to the transformation and evolution process as well as the ability of some oncogenic protein to evade CMA degradation remain to be elucidated. For this purpose, it has become essential to develop effective methods to monitor and selectively modulate CMA in humans—still a very complex challenge.

## Figures and Tables

**Table 1:** Pros and cons of the different methods used to monitor CMA.

Method	Pros	Cons
Immunoblot of LAMP-2A	- Technically simple	- Overall estimate of CMA changes
Quantification of CMA active lysosomes	- Technically simple	- Overall estimate of CMA changes - Methanol fixation
Monitoring the degradation of radiolabelled long-lived protein	- Measures CMA flux	- Involves use of radioactivity - Requires inhibition of other autophagic pathways - Measures CMA and microautophagy
Measurement of the uptake of CMA substrate by isolated lysosomes	- Reconstitution of CMA in vitro - Measures functional CMA definitively	- Requires lysosome isolation - Requires large volume of cells or tissues
Photoswitchable/photoactivable CMA reporter	- Measures CMA flux	- Overall estimate of CMA changes - Must be complemented by other methods - Difficult to distinguish surface bound from translocated substrate
GAPDH-Halo tag fluorescence-based method	- Measures CMA flux	- Overall estimate of CMA changes - Must be complemented by other methods - Measures CMA and microautophagy
KFERQ Dendra reporter	- Measures CMA flux in vitro and in vivo	- Must be complemented by other methods
